# Failure of Manganese to Protect from Shiga Toxin

**DOI:** 10.1371/journal.pone.0069823

**Published:** 2013-07-16

**Authors:** Marsha A. Gaston, Christine A. Pellino, Alison A. Weiss

**Affiliations:** Department of Molecular Genetics, Biochemistry, and Microbiology, College of Medicine, University of Cincinnati, Cincinnati, Ohio, United States of America; University of Padova, Medical School, Italy

## Abstract

Shiga toxin (Stx), the main virulence factor of Shiga toxin producing *Escherichia coli*, is a major public health threat, causing hemorrhagic colitis and hemolytic uremic syndrome. Currently, there are no approved therapeutics for these infections; however manganese has been reported to provide protection from the Stx1 variant isolated from *Shigella dysenteriae* (Stx1-S) both *in vitro* and *in vivo*. We investigated the efficacy of manganese protection from Stx1-S and the more potent Stx2a isoform, using experimental systems well-established for studying Stx: *in vitro* responses of Vero monkey kidney cells, and *in vivo* toxicity to CD-1 outbred mice. Manganese treatment at the reported therapeutic concentration was toxic to Vero cells in culture and to CD-1 mice. At lower manganese concentrations that were better tolerated, we observed no protection from Stx1-S or Stx2a toxicity. The ability of manganese to prevent the effects of Stx may be particular to certain cell lines, mouse strains, or may only be manifested at high, potentially toxic manganese concentrations.

## Introduction

Each year in the US, Shiga toxin (Stx) producing *Escherichia coli* (STEC) are responsible for over 100,000 cases of infectious diarrhea. Of these infected individuals, about 10% develop more severe sequelae such as life-threatening hemolytic uremic syndrome (HUS) [1]. The primary virulence factor, Stx, is responsible for disease symptoms. Stx is an AB_5_ toxin, comprised of a receptor binding pentameric B-subunit and an enzymatically active monomeric A-subunit that inhibits protein synthesis. There are two major antigenic forms, Stx1 and Stx2. These forms share greater than 50% amino acid identity, but do not generate cross-neutralizing antibodies. In the past, the original toxin isolated from *Shigella dysenteriae* has been referred to as Stx, the highly related form isolated from *E. coli* has been referred to Stx1, and Stx2 has been used to refer to the highly potent form isolated from *E. coli*. However, numerous polymorphic forms of Stx2 have now been described which can share over 90% amino acid identity, but vary in potency by several orders of magnitude [2]. As more variants have been sequenced, the historic nomenclature has become extremely ambiguous. To avoid confusion, we will refer to the family members as Stx1 and Stx2, and variants used in this study as Stx1-S (the variant isolated from *S. dysenteriae*) and Stx2a (the highly potent variant first isolated from EDL933). STEC can express one, or both forms of toxin. The reduced potency of Stx1 compared to Stx2a is well documented in mice [2], [3] and primates [4], [5]. Furthermore, Stx2a is more commonly associated with life-threatening human disease; the majority of cases of HUS are associated with strains that produce Stx2a [6].

Other than supportive treatment, there are currently no therapeutics for STEC infections. However, past studies have shown that pre-treatment with certain ions, including Mn^2+^, can play a protective role against Stx intoxication [7], [8]. Sandvig and Brown previously reported protection from Stx1-S in Vero (African green monkey kidney) cells and HeLa cells when incubated in the presence of high concentrations of certain ions [7]. Using protein synthesis as an assessment of Stx1-S toxicity, Sandvig and Brown show that HeLa cells and Vero cells were protected from Stx1-S when incubated in the presence of 2 mM MnCl_2_, CoCl_2_, or BaCl_2_. MgCl_2_ at 2 mM also provided protection for Vero cells but not HeLa cells. Additionally, the presence of calcium ionophores and high concentrations (140 mM) of anions SCN^−^ and SO_4_^−^ also protected these cell lines from Stx1-S. It was thus hypothesized that inhibitors of Ca^2+^ and Cl^-^ transport could protect cells from Stx1-S. However, the toxicity of the treatments themselves was not assessed.

Similarly, a recent report by Mukhopadhyay and Linstedt presents manganese as a potential treatment for Shiga toxicosis by blocking Stx1-S trafficking [8]. Proper trafficking through the cell is essential to Stx toxicity. After endocytosis, the Stx holotoxin is trafficked from early endosomes to the Golgi apparatus and endoplasmic reticulum (ER) [9], [10]. In the ER, the enzymatic A-subunit separates from the holotoxin, is processed, and released into the cytosol where it inhibits protein synthesis by cleaving a conserved adenine in 28S ribosomal RNA [11]. While the ER is the final destination of the holotoxin, trafficking through the Golgi is a required step [12]. Mukhopadhyay and Linstedt conclude that HeLa cell protection against Stx1-S toxicity in the presence of manganese is due to altered trafficking; demonstrating that pretreating HeLa cells with 500 µM MnCl_2_ diverts trafficking of the Stx B-subunit from the Golgi to lysosomes, where it was subsequently degraded [8]. When assayed using Stx1-S holotoxin, HeLa cells were protected in the presence of manganese. Moreover, the manganese treatment is reported to protect BALB/c mice from Stx1-S toxicity.

These prior studies were all performed using Stx1-S, the less potent form of the toxin. We set out to investigate if manganese would also provide protection from Stx2a. In our experimental systems, we observe that not only is manganese itself toxic at previously reported treatment doses, but manganese treatment at lower, less toxic MnCl_2_ concentrations, offers no protection from Stx either *in vitro* or *in vivo*.

## Materials and Methods

### Ethics Statement

All animal work was conducted according to relevant national and international guidelines, including the requirements of the Association for the Assessment and Accreditation of Laboratory Animal Care International as described in the Guide for the Care and Use of Laboratory Animals, Eighth Edition. Animals in this study were housed in the animal facilities at University of Cincinnati and all experiments performed according to Protocol # 08-11-18-01, approved by the University of Cincinnati Institutional Animal Care and Use Committee (IACUC). Animals were weighed daily; those appearing moribund or losing more than 20% of initial body weight were euthanized in compliance with IACUC regulations.

### Cell Lines and Materials

Luc2P Vero cells, a Vero cell line transfected to express *luc2p*, a gene for destabilized luciferase, was used for all *in vitro* toxicity assays (strain constructed as described in [13]). Cells were cultured in Modified Eagle Medium supplemented with 10% heat-inactivated fetal bovine serum, 1× penicillin/streptomycin/glutamine solution, and 1× MEM vitamins solution (sMEM) (Life Technologies, Grand Island, NY). The following were obtained from the Biodefense and Emerging Infectious Diseases Research Resources Repository, NIAID, NIH: Stx1-S (Shiga Toxin Type 1, Recombinant from Escherichia coli, NR-857) and Stx2a holotoxin (Shiga Toxin Type 2, Recombinant from Escherichia coli, NR-4478).

### *In vitro* Toxicity Assays

For manganese toxicity assays, a solution of concentrated MnCl_2_ in Tris buffered saline, pH 7.4 (TBS) was prepared and used on the same day. This solution was diluted in sMEM in white tissue culture treated microtiter plates (BD Falcon, Falkin Lakes, NJ). As a control, a set of wells containing no MnCl_2_ was included in the same plate. Luc2P Vero cells, 10^4^ per well, were added such that final MnCl_2_ concentrations ranged from 1000 µM to 2.5 µM.

Cells were incubated at 37°C with 5% CO_2_ for four hours. After this incubation, one half of the media was removed and replaced with an equal volume of TBS to mimic the experimental system of Mukhopadhyay and Linstedt [8]. Following a second four hour incubation, media was aspirated, cells were washed three times with phosphate buffered saline, pH 7.4 (PBS), and 25 µl of SuperLite luciferase substrate (Bioassay Systems, Hayward, CA) was added to each well. Light production was measured using Luminoskan Ascent (Thermo Labsystems, Helsinki, Finland).

For the *in vitro* Stx toxicity assays, MnCl_2_ was added to 10^4^ Luc2P Vero cells at final concentration of 250 µM as described above. Three sets of wells lacking MnCl_2_ were included on the same plate, as negative controls. Cells were incubated at 37°C with 5% CO_2_ for four hours. After this incubation, one half of the media was removed and replaced with purified Stx1-S or Stx2a holotoxin serially diluted in TBS. Following a second four hour incubation, cells were washed and light production was measured as described above.

Percent protein synthesis was correlated to the average light production of the control well without manganese or toxin representing 100%. A two-tailed Student’s *t* test (GraphPad Prism 5, La Jolla, CA) was used to calculate statistical differences.

### *In vivo* Toxicity Assays

Male CD-1 mice, 13–15 g (aged 22–23 days), were obtained from Charles Rivers Laboratories (Wilmington, MA). Three days after arrival, mice received either 50 mg/kg MnCl_2_ in 0.5 ml sterile water or 0.5 ml water alone intraperitoneally (IP). Within five minutes post injection of MnCl_2_ at the reported sub-lethal dose of 50 mg/kg [8], mice exhibited signs of distress and the treatment was subsequently performed with half the reported sub-lethal dose of MnCl_2_ (25 mg/kg).

Animal studies were performed as described previously by Mukhopadhyay and Linstedt [8]. Mice underwent once daily IP injections of MnCl_2_ (25 mg/kg) or water five days prior to and subsequently every day post IP challenge with a lethal dose of either Stx1-S (2000 ng) or Stx2a (7 ng) in 0.5 ml PBS, or a sham challenge of 0.5 ml PBS. On the day of Stx injections, animals received toxin and either MnCl_2_ or water IP bilaterally. Animals treated with either water alone or MnCl_2_ alone: n = 4 per group. Animals treated with water or MnCl_2_ that were challenged with Stx2a: n = 6 per group. Animals treated with water or MnCl_2_ that were challenged with Stx1-S: n = 4 per group.

## Results

### Manganese Toxicity in Vero Cells

**Figure 1:**
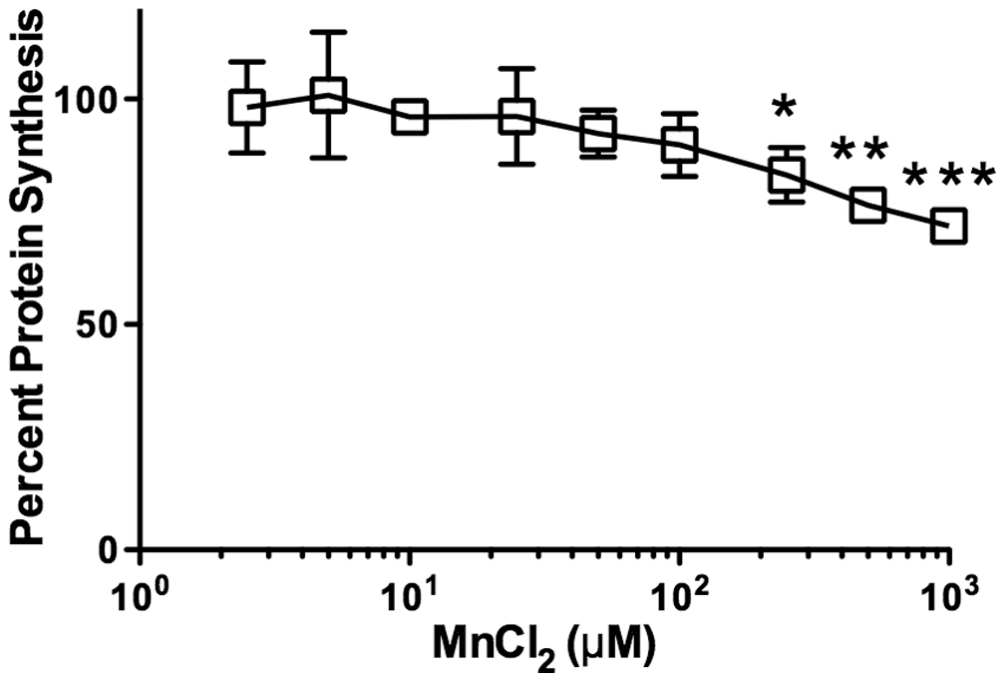
Manganese exposure results in decreased protein synthesis in Vero cells. Percent protein synthesis of Vero cells incubated in the presence of various concentrations of MnCl_2_ for 4 hours followed by an additional 4 hour incubation at half of the MnCl_2_ concentration. One hundred percent protein synthesis was determined from Vero cells incubated in the absence of any exogenous MnCl_2_. All points were assayed in triplicate and error bars indicate standard deviation. Two-tailed Student’s *t* test was used to assess statistical significance when compared to the no manganese control (* *P*<0.05, ** *P*<0.005, *** *P*<0.0005).

A previously described cell line, Luc2P Vero African green monkey kidney epithelial cells, was used for *in vitro* toxicity assays [13]. These cells express Luc2P, a destabilized form of luciferase that is rapidly targeted for proteasomal degradation and does not accumulate in the cell. Thus, luciferase activity can be directly correlated to the rate of protein synthesis. In addition, the ED_50_ for protein synthesis inhibition was shown to correlate well with traditional assays measuring cellular metabolic activity at 3 days post toxin treatment. To assess the concentrations at which MnCl_2_ is toxic to Vero cells, luciferase activity was measured after Luc2P cells were incubated for four hours in the presence MnCl_2_ ranging in concentration from 0 µM to 1000 µM, then for an additional four hours at half of the initial MnCl_2_. This methodology was intended to be similar to that used previously to assess Mn^2+^ protection from Stx1-S toxicity in HeLa cells [8]. Cells incubated in initial MnCl_2_ concentrations ranging from 250 µM to 1000 µM demonstrated significantly lower rates of protein synthesis as compared to the no manganese control (Fig. 1). At 500 µM MnCl_2_, the concentration previously used with HeLa cells with no reported toxicity, Vero cells demonstrated approximately a 25% decrease in protein synthesis compared to cells with no exogenous Mn^2+^ added (*P* = 0.0014). Approximately a 17% decrease in protein synthesis was observed at 250 µM MnCl_2_ (*P* = 0.0134).

### Manganese Offers No Protection from Stx Toxicity in Vero Cells

**Figure 2:**
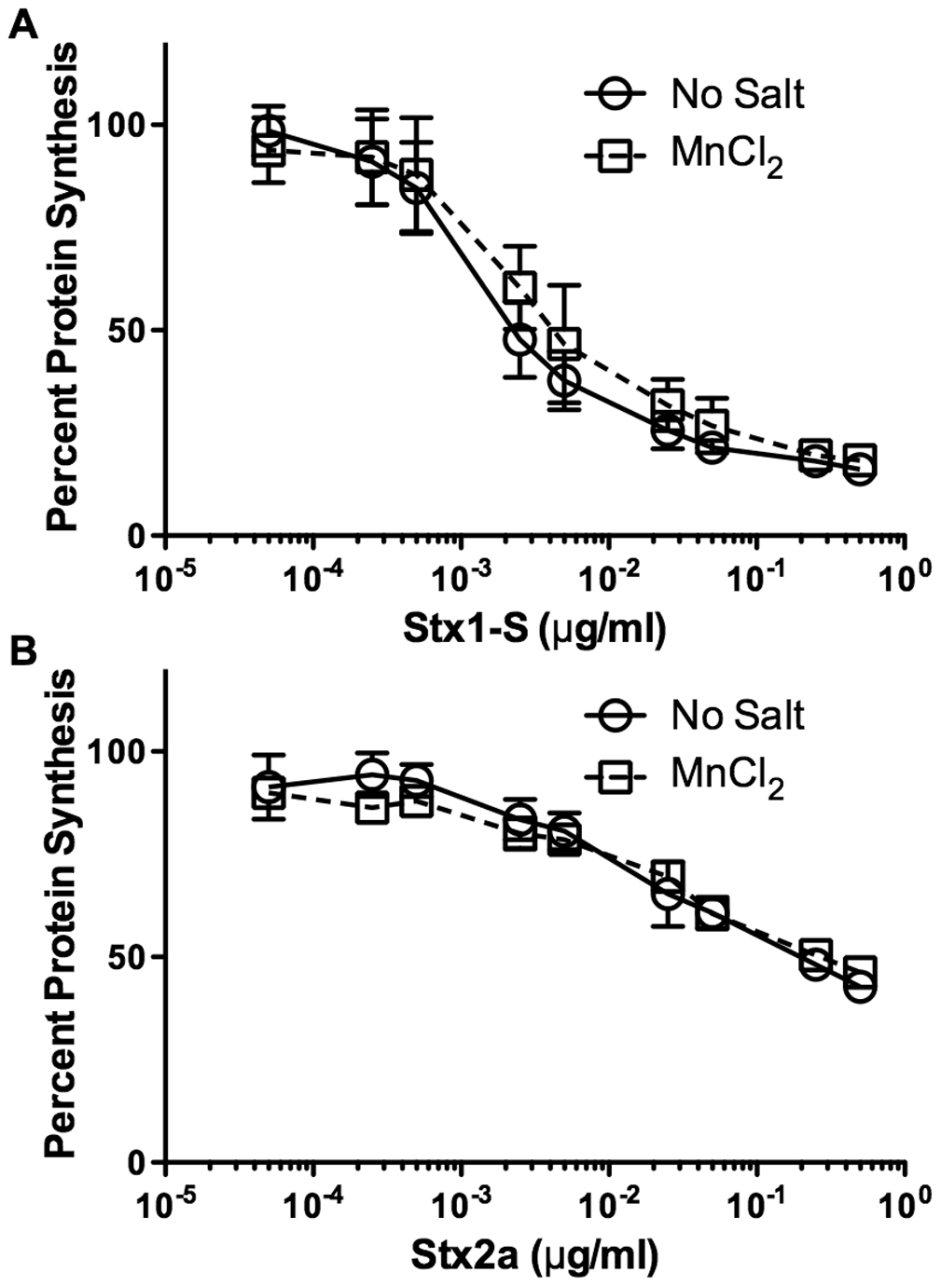
Manganese pretreatment offers no protection against the effects of Stx in Vero cells. One hundred percent protein synthesis corresponds to cells incubated with no exogenous MnCl_2_ and no toxin. All points were assayed in triplicate and error bars indicate standard deviation. (**A, B**) Percent protein synthesis of Vero cells incubated in the presence or absence of 250 µM MnCl_2_ for 4 hours followed by an additional 4 hour incubation at 125 µM MnCl_2_ with increasing concentrations of either (**A**) Stx1-S or (**B**) Stx2a.

To assess the protective effects of manganese on Luc2P Vero cells from Stx-mediated toxicity, cells were preincubated for four hours in the presence or absence of 250 µM MnCl_2_, then incubated for an additional four hours in 125 µM MnCl_2_ in the presence of various dilutions (ranging from 5×10^−5^ to 5×10^−1^ µg/ml as indicated in Fig. 2) of purified Stx1-S or Stx2a. While still toxic, this manganese concentration was chosen to be high enough to see a protective effect yet be minimally toxic itself. Luciferase activity was measured at the end of this second four hour incubation. One hundred percent protein synthesis was defined as the amount of light measured from cells incubated in sMEM and TBS without either MnCl_2_ or toxin (Fig. 2). The effective dose for 50% inhibition of protein synthesis (ED_50_) for Stx1-S in the absence of manganese was determined to be 2.15 ng/ml, while the value for Stx2a was determined to be 192 ng/ml. Both of these values are much higher than previously reported values for Stx1-S (63 pg/ml) and Stx2a (461 pg/ml) [13]. However, in the previous report, protein synthesis was measured four hours after the cells were suspended in fresh media, while in this assay, protein synthesis is measured eight hours after the cells were suspended in fresh media. Decreased metabolic activity could account for the increased resistance. Alternatively, toxin susceptibility has been shown to be influenced by cell cycle; Vero cells are most susceptible at the G1/S boundary, and the proportion of cells at this stage in the cell cycle could be reduced in the longer assay [14]. Moreover, in the previous report cells are exposed to toxin before they adhere to the plate, whereas they are adherent in this assay at the time of toxin addition. It is possible that the increased toxicity reported previously is due to increased cell surface area of non-adhered cells for toxin binding. The ED_50_ for Stx1-S in the presence of MnCl_2_ (4.06 ng/ml) was slightly increased compared to the cells in the absence of MnCl_2_ (2.15 ng/ml) (Fig. 2A), but this difference was not statistically significant. Similarly, a manganese treatment provided no significant protection for cells exposed to Stx2a (Fig. 2B), and the ED_50_ values appeared to be identical.

### Stx1-S and Stx2a are Lethal in Mice Treated with Manganese

**Figure 3:**
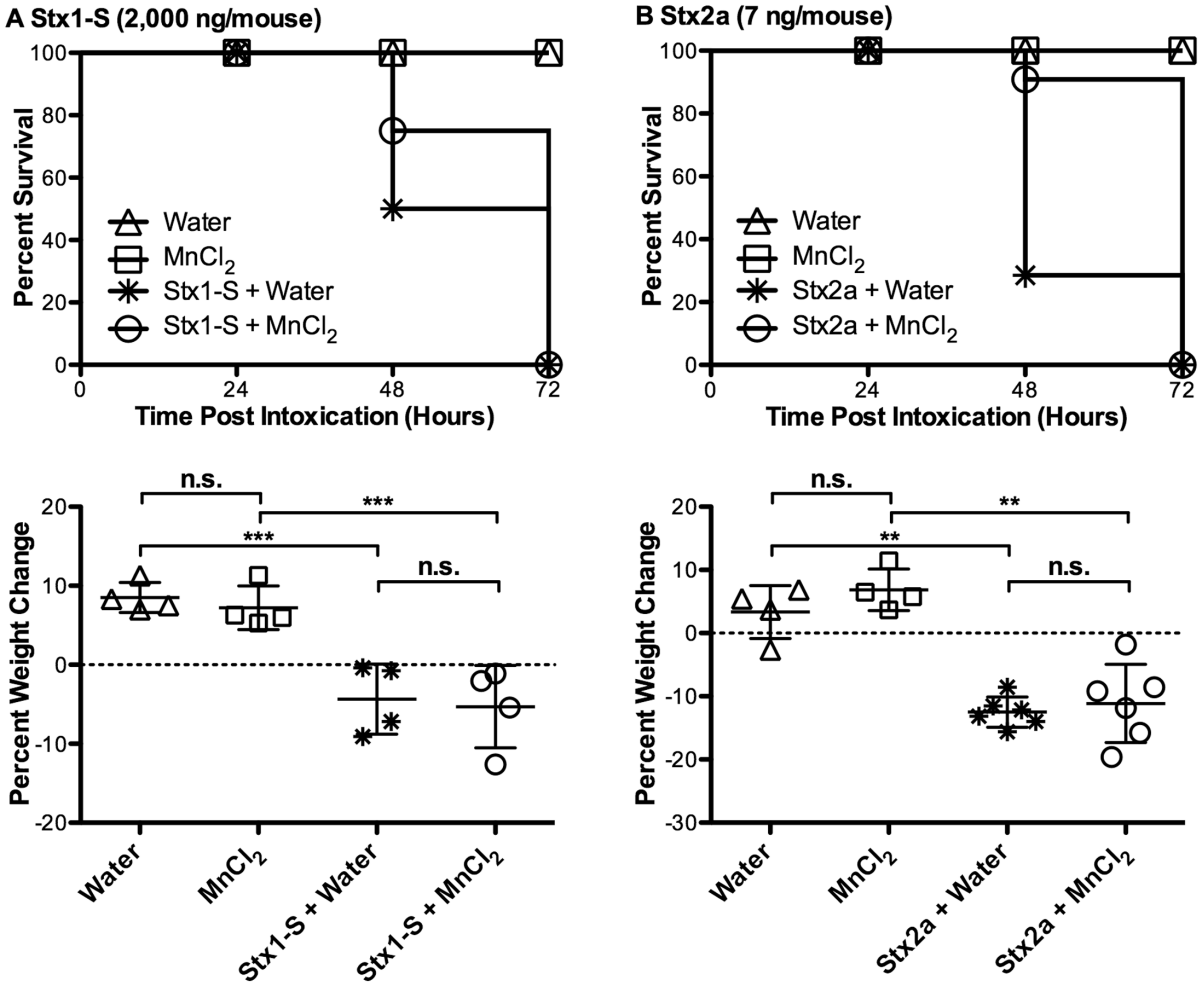
Treatment with manganese does not protect CD-1 mice from (A) Stx1-S or (B) Stx2a. **Top panels**, survival curves from mice treated with Water, MnCl_2_, Stx+Water, or Stx+MnCl_2_ (Water, MnCl_2_, Stx1-S+Water, Stx1-S+MnCl_2_: n = 4 per group; Stx2a+Water, Stx2a+MnCl_2_: n = 6 per group). **Bottom panels**, average percentage weight change at 48 hours post challenge. Two-tailed Student’s *t* test was used to assess statistical significance (n.s. no statistical significance, ** *P*<0.005, *** *P*<0.0005).

We evaluated MnCl_2_ protection from Stx1-S and Stx2a to outbred CD-1 mice. CD-1 mice were more sensitive to MnCl_2_ than BALB/c mice. Within five minutes post-injection of MnCl_2_ at the reported dose of 50 mg/kg, mice exhibited signs of distress including: hunching, isolation in the corner of cages and temporary tremor. These signs were not reported for BALB/c mice [8]. We thus used half the reported dose of MnCl_2_ for our *in vivo* Stx toxicity assays. CD-1 mice were injected daily with MnCl_2_ (25 mg/kg) five days prior to and subsequently every day post challenge (IP) with a lethal dose of Stx or sham dose of PBS. In previous studies, the LD_50_ for Stx1-S was determined to be greater than 1,000 ng, and the LD_50_ for Stx2a was determined to be about 6 ng [2]. To ensure lethality, mice were challenged IP with 2000 ng of Stx1-S and 7 ng of Stx2a. No increase in survival was observed compared to mice treated with water alone (Fig. 3A and B). Additionally, percent weight change can be used to assess morbidity in animals challenged with Stx. No difference in morbidity was seen between treatment groups in animals challenged with Stx, as exhibited by no significant difference in weight loss at 48 hours post challenge. Percent weight change, however, is significantly different between non-challenge and Stx challenge treatment groups (Fig. 3A and B).

## Discussion

No therapeutics are available for STEC infections, and we were greatly intrigued by studies reporting that manganese could protect from Stx1-S mediated toxicity to HeLa cells *in vitro* and BALB/c mice *in vivo* [7], [8]. As these studies only investigated protection from the less potent Stx1-S, we investigated the potential of manganese to protect from both Stx1-S and the more potent Stx2a in experimental systems well-established for assessing Stx toxicity: *in vitro*, using Vero monkey kidney epithelial cells, and *in vivo*, using outbred CD-1 mice.

Mukhopadhyay and Linstedt reported that manganese protects cells *in vitro* from Stx1-S [8]. However, in our studies, we did not observe manganese protection from either Stx1-S or Stx2a using an experimental system that differed from those of Mukhopadhyay and Linstedt in several respects. First, while they used HeLa cells engineered to be sensitive to Stx by up-regulating expression of the Stx receptor, we used Vero cells, which are naturally sensitive to Stx. Mukhopadhyay and Linstedt assessed cellular health by examining mitochondrial dehydrogenase activity using methylthiazolyldiphenyl-tetrazolium bromide (MTT); alternatively, we measured the rate of protein synthesis. As Stx targets protein synthesis through inactivation of the ribosome, and does not directly target mitochondrial respiration, our methodologies are a more direct assessment of protection from Stx. Finally, we assessed protection from Stx at lower concentrations of manganese because we found that the manganese treatment, in the absence of Stx, inhibited protein synthesis.

*In vivo,* Mukhopadhyay and Linstedt reported that the manganese doses up to 50 mg/kg did not cause stress in the BALB/c mice [8]. However, this dose of MnCl_2_ caused systemic symptoms within 5 minutes in the outbred CD-1 mice used in our study. We used manganese at a lower dose (25 mg/kg), which was also reported to confer protection by Mukhopadhyay and Linstedt. This dose did not appear to cause stress to the CD-1 mice, but failed to confer protection from toxin-mediated death from either Stx1-S or the more medically relevant Stx2a. While Mukhopadhyay and Linstedt reported that manganese is cleared from the mice within hours, they show protection against intoxication with a once daily injection of Mn^2+^ five days prior to and everyday post challenge with Stx1-S, at approximately 500 ng Stx1-S per mouse [8]. Using this same model with CD-1 mice, in our study all of the mice died on either day 2 or day 3 post-challenge. No difference in body weight was seen at 48 hours after challenge, suggesting that increased time to death does not reflect protection.

The use of different experimental systems could account for the failure to reproduce the reported results. In human disease, Stx is known to target three different cell types which naturally express globotriaosylceramide (Gb3), the glycolipid receptor for Stx: kidney cells, endothelial cells and neurons [1], [15]-[17]. The female reproductive tract, where HeLa cells originated, has not been reported to be targeted by Stx. HeLa are likely susceptible to Stx because upregulation of Gb3 expression is common in cancer cells [18]. Nevertheless, HeLa cells are still more resistant to Stx than Vero cells. Mukhopadhyay and Linstedt used HeLa cells transfected to express Gb3 synthase, to increase expression of the Gb3 receptor, and demonstrated that Stx resistance is due to altered intracellular trafficking in HeLa cells [8]. However, it is known that Stx uses different pathways to enter cells [19], and it is possible that manganese does not alter Stx trafficking in its natural target cells, including kidney cells.

MnCl_2_ was also reported to protect BALB/c mice from Stx1-S [8]. We did not observe manganese protection from either Stx1-S or Stx2a in the outbred CD-1 mouse line. BALB/c mice are null mutants for Slc11a1 (formally Nramp1), an H^+^/divalent cation antiporter expressed by phagocytes with a high affinity for Mn^2+^ [20]. It is not clear if this genetic mutation could have been a factor in the observed protection against Stx1-S, but outbred CD-1 mice are likely to more closely reflect normal human physiologic responses to Mn^2+^. In addition, Stx2a, not Stx1-S, is most associated with development of fatal human disease, and the failure to observe protection form Stx2a is significant when considering treatment of human disease.

Manganese is an essential trace mineral that is used as a cofactor by enzymes that prevent oxidative stress (superoxide dismutase) [21], to detoxify byproducts of amino acid metabolism in the liver (arginase) [22], and function in collagen production (prolidase) [23]. However, manganese overexposure can be toxic, especially to the brain, resulting in permanent neurodegenerative disorders [24]. The most notorious of the conditions caused by manganese overexposure is manganism, the symptoms of which mimic those of Parkinson’s disease. Excess manganese is also implicated in decreased fertility [25], decreased sperm count and motility [26], [27], fetal skeletal development manifestations and fetal death [28], [29], and liver toxicity [30], [31]. The potential for toxicity of the treatment itself raises serious concerns whether manganese can be used to treat STEC infections. The current suggested daily allowances of manganese is 0.14 mg/kg/day, or about 10 mg/day for adults, as determined by the Environmental Protection Agency Reference Dose for Chronic Oral Exposure based on central nervous system effects in adults [32]. The US National Research Council Estimated Safe and Adequate Daily Dietary Intake suggests that 5 mg manganese per day for children and adults 10 years and older is sufficient daily intake [33]. Considering the manganese dose administered to BALB/c mice that conferred protection from Stx1-S, a daily therapeutic dose for an adult weighing approximately 70 kg would be approximately 3,500 mg, 350 times greater than the suggested daily allowance in adults.

In summary, currently there are no therapeutics for Stx-mediated toxicity, and our studies suggest manganese holds little promise as a therapeutic candidate.
